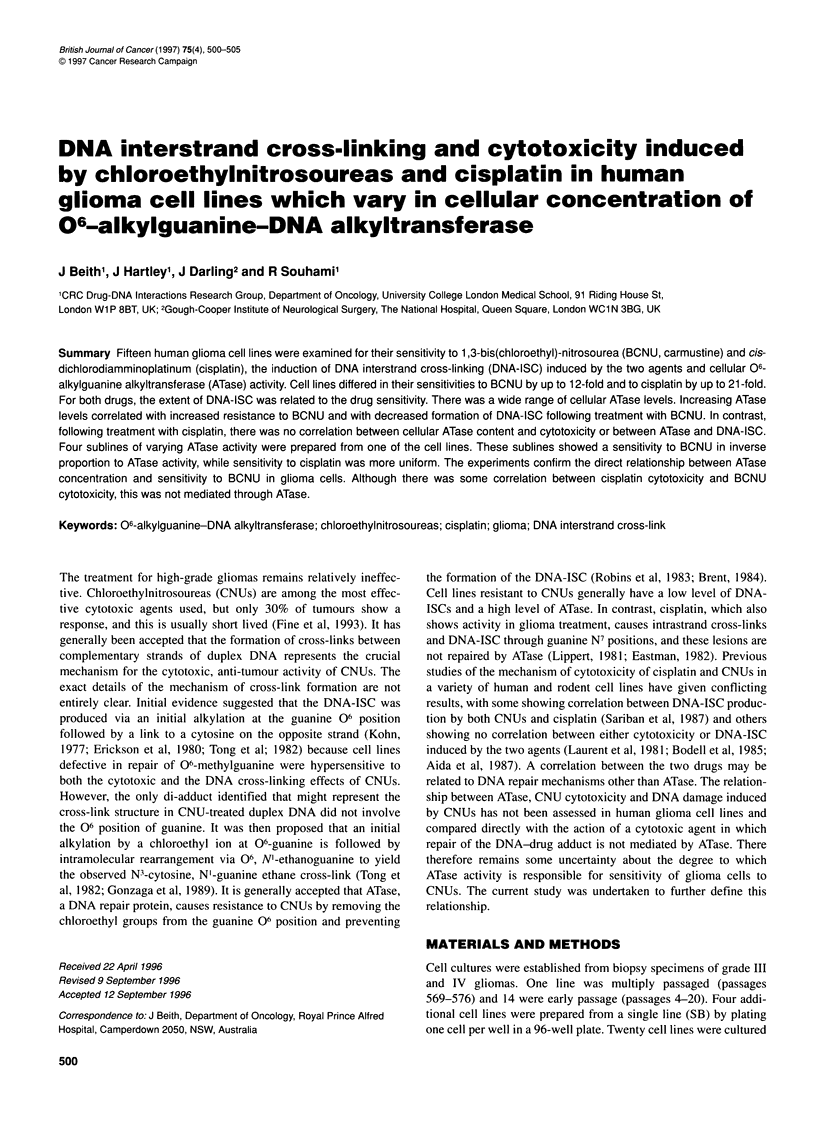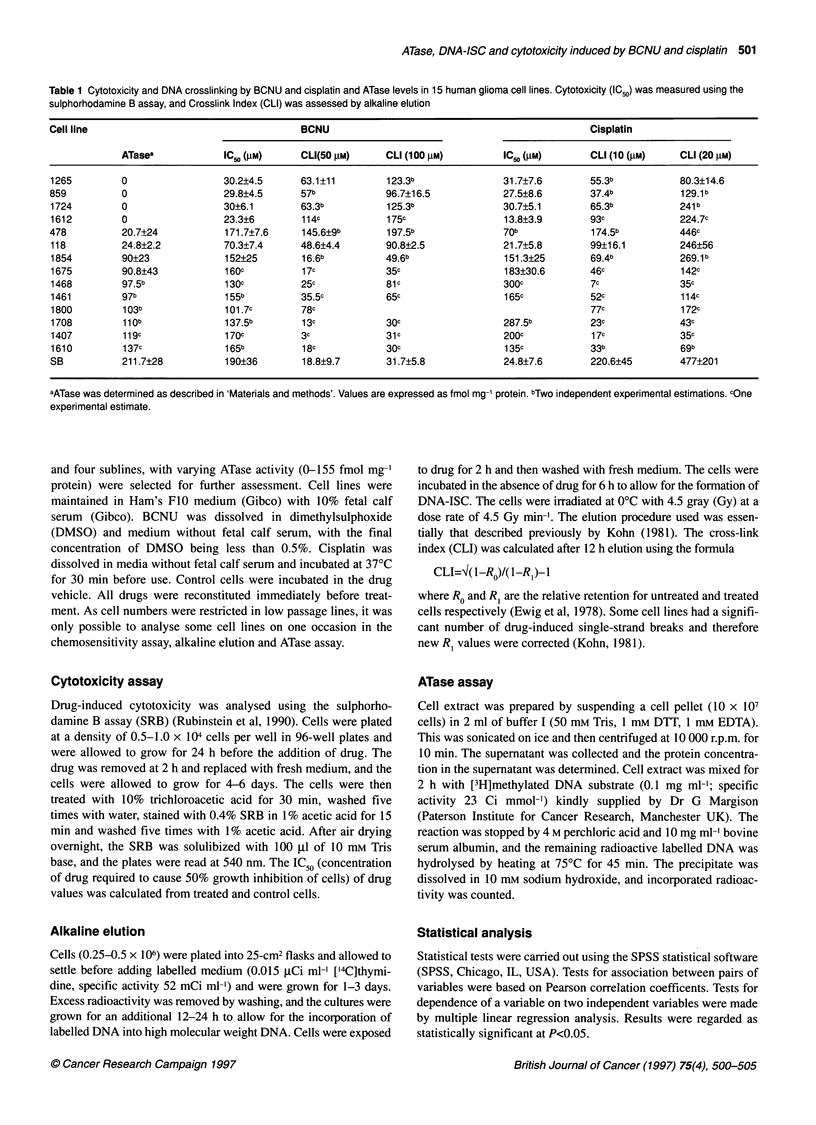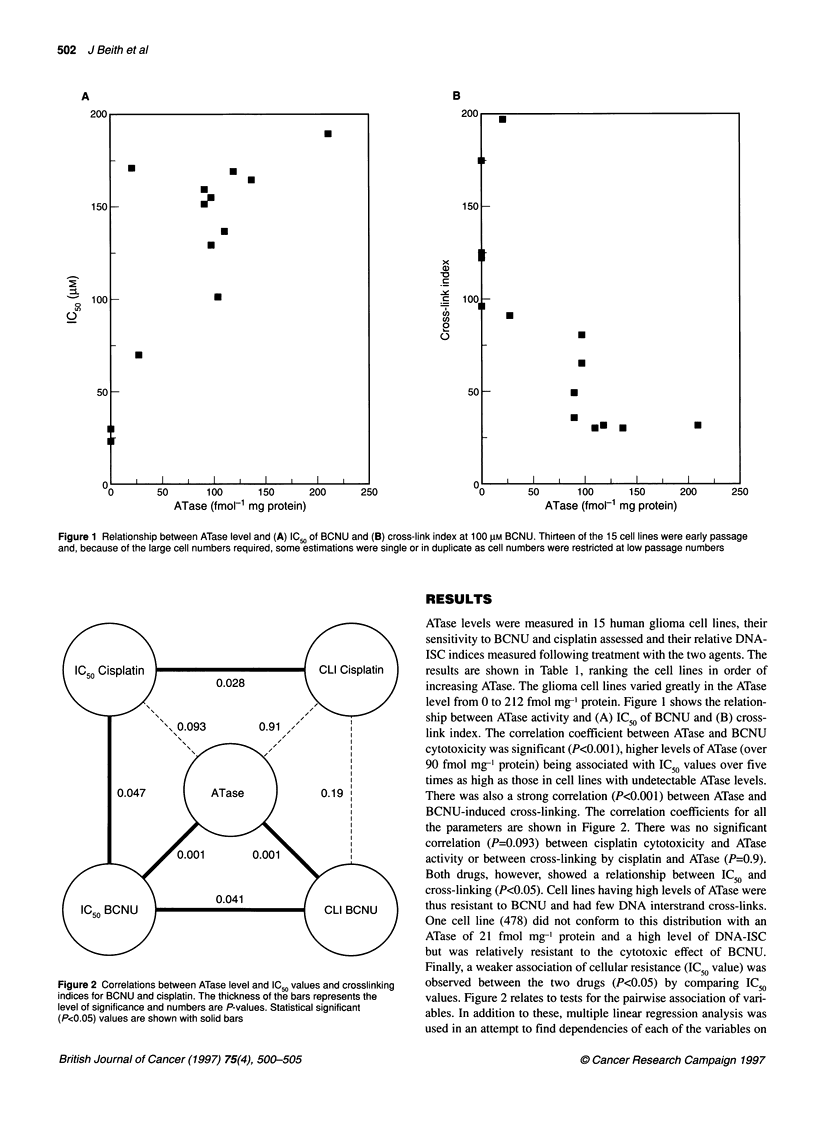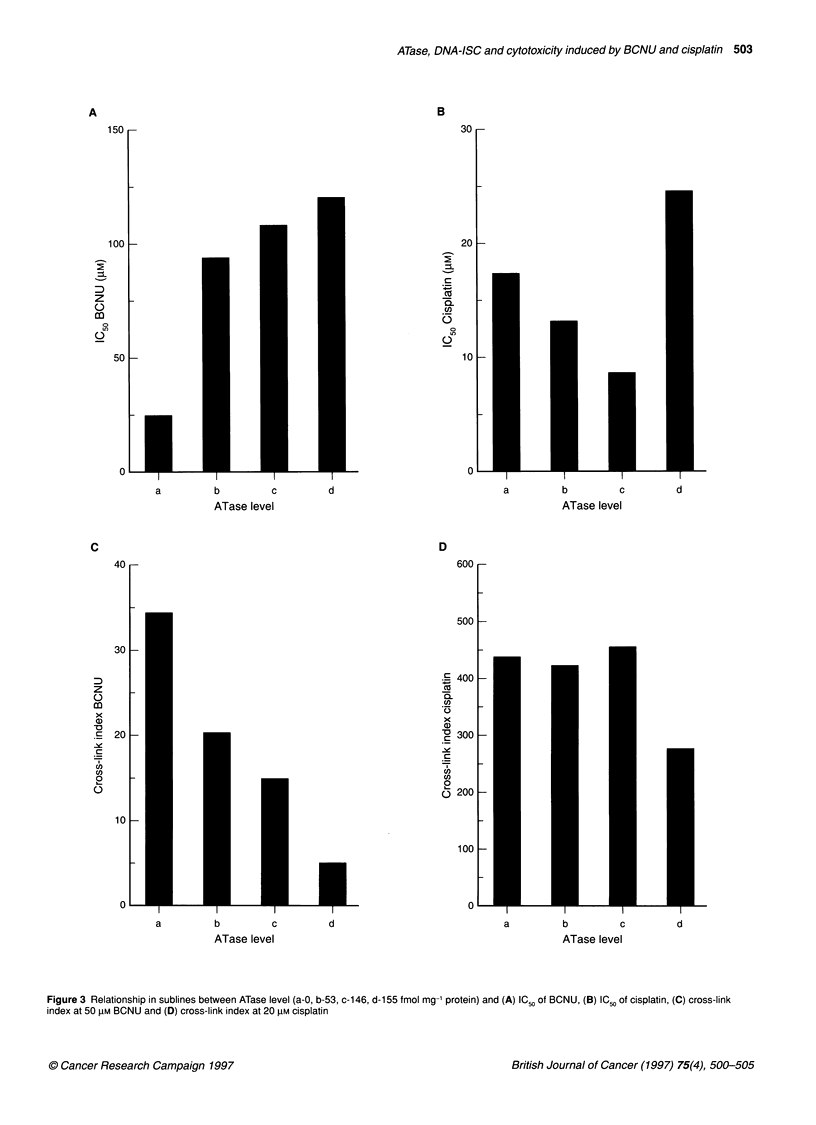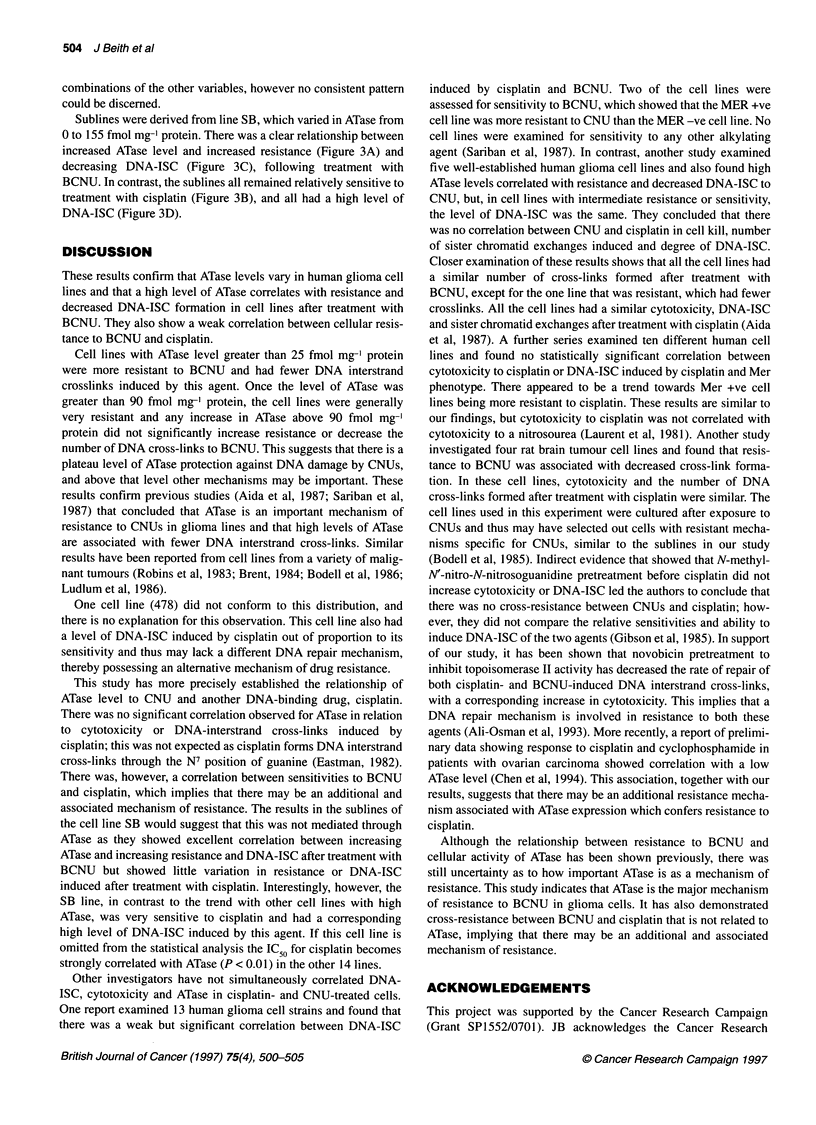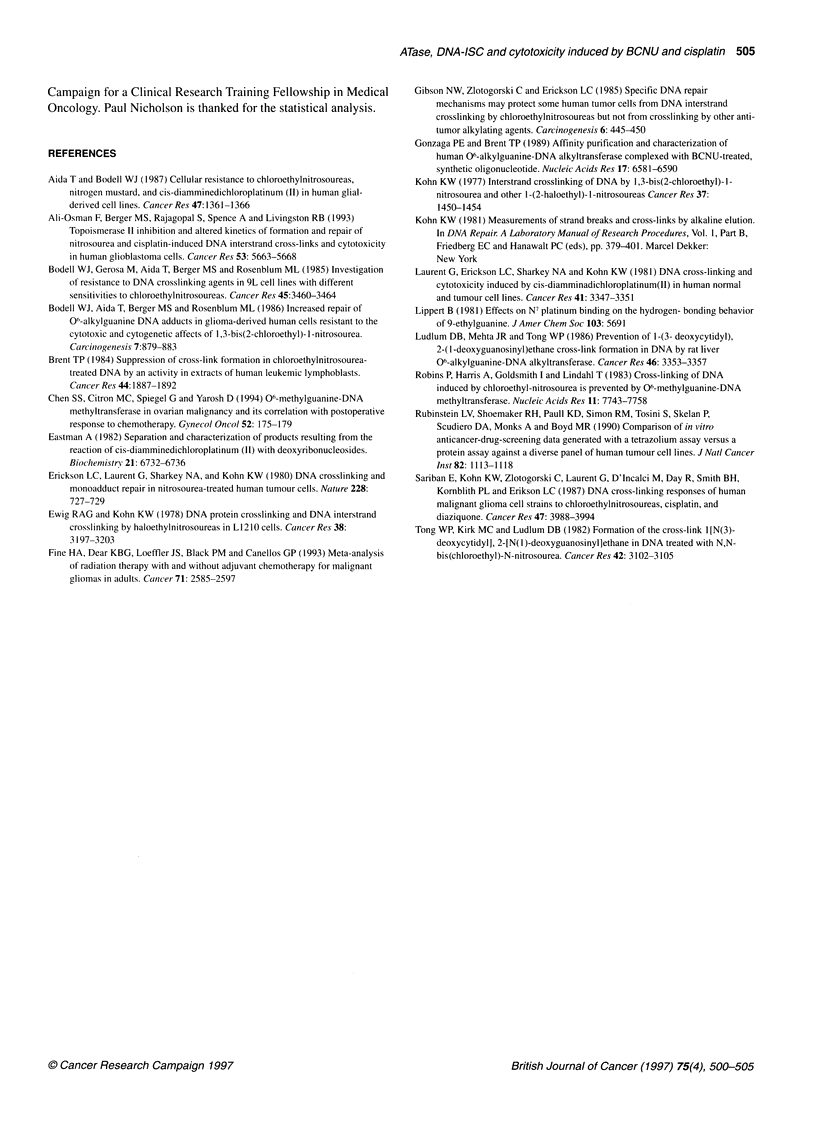# DNA interstrand cross-linking and cytotoxicity induced by chloroethylnitrosoureas and cisplatin in human glioma cell lines which vary in cellular concentration of O6-alkylguanine-DNA alkyltransferase.

**DOI:** 10.1038/bjc.1997.87

**Published:** 1997

**Authors:** J. Beith, J. Hartley, J. Darling, R. Souhami

**Affiliations:** Department of Oncology, University College London Medical School, UK.

## Abstract

Fifteen human glioma cell lines were examined for their sensitivity to 1,3-bis(chloroethyl)-nitrosourea (BCNU, carmustine) and cis-dichlorodiamminoplatinum (cisplatin), the induction of DNA interstrand cross-linking (DNA-ISC) induced by the two agents and cellular O6-alkylguanine alkyltransferase (ATase) activity. Cell lines differed in their sensitivities to BCNU by up to 12-fold and to cisplatin by up to 21-fold. For both drugs, the extent of DNA-ISC was related to the drug sensitivity. There was a wide range of cellular ATase levels. Increasing ATase levels correlated with increased resistance to BCNU and with decreased formation of DNA-ISC following treatment with BCNU. In contrast, following treatment with cisplatin, there was no correlation between cellular ATase content and cytotoxicity or between ATase and DNA-ISC. Four sublines of varying ATase activity were prepared from one of the cell lines. These sublines showed a sensitivity to BCNU in inverse proportion to ATase activity, while sensitivity to cisplatin was more uniform. The experiments confirm the direct relationship between ATase concentration and sensitivity to BCNU in glioma cells. Although there was some correlation between cisplatin cytotoxicity and BCNU cytotoxicity, this was not mediated through ATase.


British Journal of Cancer (1997) 75(4), 500–505
© 1997 Cancer Research Campaign

### DNA interstrand cross-linking and cytotoxicity induced by chloroethylnitrosoureas and cisplatin in human glioma cell lines which vary in cellular concentration of $O^6$-alkylguanine–DNA alkyltransferase

**J Beith[1], J Hartley[1], J Darling[2] and R Souhami[1]**

[1]CRC Drug-DNA Interactions Research Group, Department of Oncology, University College London Medical School, 91 Riding House St, London W1P 8BT, UK; [2]Gough-Cooper Institute of Neurological Surgery, The National Hospital, Queen Square, London WC1N 3BG, UK

**Summary** Fifteen human glioma cell lines were examined for their sensitivity to 1,3-bis(chloroethyl)-nitrosourea (BCNU, carmustine) and *cis*-dichlorodiamminoplatinum (cisplatin), the induction of DNA interstrand cross-linking (DNA-ISC) induced by the two agents and cellular $O^6$-alkylguanine alkyltransferase (ATase) activity. Cell lines differed in their sensitivities to BCNU by up to 12-fold and to cisplatin by up to 21-fold. For both drugs, the extent of DNA-ISC was related to the drug sensitivity. There was a wide range of cellular ATase levels. Increasing ATase levels correlated with increased resistance to BCNU and with decreased formation of DNA-ISC following treatment with BCNU. In contrast, following treatment with cisplatin, there was no correlation between cellular ATase content and cytotoxicity or between ATase and DNA-ISC. Four sublines of varying ATase activity were prepared from one of the cell lines. These sublines showed a sensitivity to BCNU in inverse proportion to ATase activity, while sensitivity to cisplatin was more uniform. The experiments confirm the direct relationship between ATase concentration and sensitivity to BCNU in glioma cells. Although there was some correlation between cisplatin cytotoxicity and BCNU cytotoxicity, this was not mediated through ATase.

**Keywords:** $O^6$-alkylguanine–DNA alkyltransferase; chloroethylnitrosoureas; cisplatin; glioma; DNA interstrand cross-link

The treatment for high-grade gliomas remains relatively ineffective. Chloroethylnitrosoureas (CNUs) are among the most effective cytotoxic agents used, but only 30% of tumours show a response, and this is usually short lived (Fine et al, 1993). It has generally been accepted that the formation of cross-links between complementary strands of duplex DNA represents the crucial mechanism for the cytotoxic, anti-tumour activity of CNUs. The exact details of the mechanism of cross-link formation are not entirely clear. Initial evidence suggested that the DNA-ISC was produced via an initial alkylation at the guanine $O^6$ position followed by a link to a cytosine on the opposite strand (Kohn, 1977; Erickson et al, 1980; Tong et al; 1982) because cell lines defective in repair of $O^6$-methylguanine were hypersensitive to both the cytotoxic and the DNA cross-linking effects of CNUs. However, the only di-adduct identified that might represent the cross-link structure in CNU-treated duplex DNA did not involve the $O^6$ position of guanine. It was then proposed that an initial alkylation by a chloroethyl ion at $O^6$-guanine is followed by intramolecular rearrangement via $O^6$, $N^1$-ethanoguanine to yield the observed $N^3$-cytosine, $N^1$-guanine ethane cross-link (Tong et al, 1982; Gonzaga et al, 1989). It is generally accepted that ATase, a DNA repair protein, causes resistance to CNUs by removing the chloroethyl groups from the guanine $O^6$ position and preventing the formation of the DNA-ISC (Robins et al, 1983; Brent, 1984). Cell lines resistant to CNUs generally have a low level of DNA-ISCs and a high level of ATase. In contrast, cisplatin, which also shows activity in glioma treatment, causes intrastrand cross-links and DNA-ISC through guanine $N^7$ positions, and these lesions are not repaired by ATase (Lippert, 1981; Eastman, 1982). Previous studies of the mechanism of cytotoxicity of cisplatin and CNUs in a variety of human and rodent cell lines have given conflicting results, with some showing correlation between DNA-ISC production by both CNUs and cisplatin (Sariban et al, 1987) and others showing no correlation between either cytotoxicity or DNA-ISC induced by the two agents (Laurent et al, 1981; Bodell et al, 1985; Aida et al, 1987). A correlation between the two drugs may be related to DNA repair mechanisms other than ATase. The relationship between ATase, CNU cytotoxicity and DNA damage induced by CNUs has not been assessed in human glioma cell lines and compared directly with the action of a cytotoxic agent in which repair of the DNA–drug adduct is not mediated by ATase. There therefore remains some uncertainty about the degree to which ATase activity is responsible for sensitivity of glioma cells to CNUs. The current study was undertaken to further define this relationship.

### MATERIALS AND METHODS

Cell cultures were established from biopsy specimens of grade III and IV gliomas. One line was multiply passaged (passages 569–576) and 14 were early passage (passages 4–20). Four additional cell lines were prepared from a single line (SB) by plating one cell per well in a 96-well plate. Twenty cell lines were cultured

*Received 22 April 1996*
*Revised 9 September 1996*
*Accepted 12 September 1996*

*Correspondence to:* J Beith, Department of Oncology, Royal Prince Alfred Hospital, Camperdown 2050, NSW, Australia

**Table 1** Cytotoxicity and DNA crosslinking by BCNU and cisplatin and ATase levels in 15 human glioma cell lines. Cytotoxicity ($IC_{50}$) was measured using the sulphorhodamine B assay, and Crosslink Index (CLI) was assessed by alkaline elution

| Cell line | | BCNU | | | Cisplatin | | |
|---|---|---|---|---|---|---|---|
| | ATase[a] | $IC_{50}$ (μM) | CLI(50 μM) | CLI (100 μM) | $IC_{50}$ (μM) | CLI (10 (μM) | CLI (20 μM) |
| 1265 | 0 | 30.2±4.5 | 63.1±11 | 123.3[b] | 31.7±7.6 | 55.3[b] | 80.3±14.6 |
| 859 | 0 | 29.8±4.5 | 57[b] | 96.7±16.5 | 27.5±8.6 | 37.4[b] | 129.1[b] |
| 1724 | 0 | 30±6.1 | 63.3[b] | 125.3[b] | 30.7±5.1 | 65.3[b] | 241[b] |
| 1612 | 0 | 23.3±6 | 114[c] | 175[c] | 13.8±3.9 | 93[c] | 224.7[c] |
| 478 | 20.7±24 | 171.7±7.6 | 145.6±9[b] | 197.5[b] | 70[b] | 174.5[b] | 446[c] |
| 118 | 24.8±2.2 | 70.3±7.4 | 48.6±4.4 | 90.8±2.5 | 21.7±5.8 | 99±16.1 | 246±56 |
| 1854 | 90±23 | 152±25 | 16.6[b] | 49.6[b] | 151.3±25 | 69.4[b] | 269.1[b] |
| 1675 | 90.8±43 | 160[c] | 17[c] | 35[c] | 183±30.6 | 46[c] | 142[c] |
| 1468 | 97.5[b] | 130[c] | 25[c] | 81[c] | 300[c] | 7[c] | 35[c] |
| 1461 | 97[b] | 155[b] | 35.5[c] | 65[c] | 165[c] | 52[c] | 114[c] |
| 1800 | 103[b] | 101.7[c] | 78[c] | | | 77[c] | 172[c] |
| 1708 | 110[b] | 137.5[b] | 13[c] | 30[c] | 287.5[b] | 23[c] | 43[c] |
| 1407 | 119[c] | 170[c] | 3[c] | 31[c] | 200[c] | 17[c] | 35[c] |
| 1610 | 137[c] | 165[b] | 18[c] | 30[c] | 135[c] | 33[b] | 69[b] |
| SB | 211.7±28 | 190±36 | 18.8±9.7 | 31.7±5.8 | 24.8±7.6 | 220.6±45 | 477±201 |

[a]ATase was determined as described in 'Materials and methods'. Values are expressed as fmol $mg^{-1}$ protein. [b]Two independent experimental estimations. [c]One experimental estimate.

and four sublines, with varying ATase activity (0–155 fmol $mg^{-1}$ protein) were selected for further assessment. Cell lines were maintained in Ham's F10 medium (Gibco) with 10% fetal calf serum (Gibco). BCNU was dissolved in dimethylsulphoxide (DMSO) and medium without fetal calf serum, with the final concentration of DMSO being less than 0.5%. Cisplatin was dissolved in media without fetal calf serum and incubated at 37°C for 30 min before use. Control cells were incubated in the drug vehicle. All drugs were reconstituted immediately before treatment. As cell numbers were restricted in low passage lines, it was only possible to analyse some cell lines on one occasion in the chemosensitivity assay, alkaline elution and ATase assay.

### Cytotoxicity assay

Drug-induced cytotoxicity was analysed using the sulphorhodamine B assay (SRB) (Rubinstein et al, 1990). Cells were plated at a density of 0.5–1.0 × $10^4$ cells per well in 96-well plates and were allowed to grow for 24 h before the addition of drug. The drug was removed at 2 h and replaced with fresh medium, and the cells were allowed to grow for 4–6 days. The cells were then treated with 10% trichloroacetic acid for 30 min, washed five times with water, stained with 0.4% SRB in 1% acetic acid for 15 min and washed five times with 1% acetic acid. After air drying overnight, the SRB was solulibized with 100 μl of 10 mM Tris base, and the plates were read at 540 nm. The $IC_{50}$ (concentration of drug required to cause 50% growth inhibition of cells) of drug values was calculated from treated and control cells.

### Alkaline elution

Cells (0.25–0.5 × $10^6$) were plated into 25-$cm^2$ flasks and allowed to settle before adding labelled medium (0.015 μCi $ml^{-1}$ [$^{14}$C]thymidine, specific activity 52 mCi $ml^{-1}$) and were grown for 1–3 days. Excess radioactivity was removed by washing, and the cultures were grown for an additional 12–24 h to allow for the incorporation of labelled DNA into high molecular weight DNA. Cells were exposed to drug for 2 h and then washed with fresh medium. The cells were incubated in the absence of drug for 6 h to allow for the formation of DNA-ISC. The cells were irradiated at 0°C with 4.5 gray (Gy) at a dose rate of 4.5 Gy $min^{-1}$. The elution procedure used was essentially that described previously by Kohn (1981). The cross-link index (CLI) was calculated after 12 h elution using the formula

$$CLI=\sqrt{(1-R_0)/(1-R_1)}-1$$

where $R_0$ and $R_1$ are the relative retention for untreated and treated cells respectively (Ewig et al, 1978). Some cell lines had a significant number of drug-induced single-strand breaks and therefore new $R_1$ values were corrected (Kohn, 1981).

### ATase assay

Cell extract was prepared by suspending a cell pellet (10 × $10^7$ cells) in 2 ml of buffer I (50 mM Tris, 1 mM DTT, 1 mM EDTA). This was sonicated on ice and then centrifuged at 10 000 r.p.m. for 10 min. The supernatant was collected and the protein concentration in the supernatant was determined. Cell extract was mixed for 2 h with [$^3$H]methylated DNA substrate (0.1 mg $ml^{-1}$; specific activity 23 Ci $mmol^{-1}$) kindly supplied by Dr G Margison (Paterson Institute for Cancer Research, Manchester UK). The reaction was stopped by 4 M perchloric acid and 10 mg $ml^{-1}$ bovine serum albumin, and the remaining radioactive labelled DNA was hydrolysed by heating at 75°C for 45 min. The precipitate was dissolved in 10 mM sodium hydroxide, and incorporated radioactivity was counted.

### Statistical analysis

Statistical tests were carried out using the SPSS statistical software (SPSS, Chicago, IL, USA). Tests for association between pairs of variables were based on Pearson correlation coefficents. Tests for dependence of a variable on two independent variables were made by multiple linear regression analysis. Results were regarded as statistically significant at $P<0.05$.

© Cancer Research Campaign 1997

**Figure 1** Relationship between ATase level and (**A**) $IC_{50}$ of BCNU and (**B**) cross-link index at 100 μM BCNU. Thirteen of the 15 cell lines were early passage and, because of the large cell numbers required, some estimations were single or in duplicate as cell numbers were restricted at low passage numbers

**Figure 2** Correlations between ATase level and $IC_{50}$ values and crosslinking indices for BCNU and cisplatin. The thickness of the bars represents the level of significance and numbers are *P*-values. Statistical significant ($P$<0.05) values are shown with solid bars

### RESULTS

ATase levels were measured in 15 human glioma cell lines, their sensitivity to BCNU and cisplatin assessed and their relative DNA-ISC indices measured following treatment with the two agents. The results are shown in Table 1, ranking the cell lines in order of increasing ATase. The glioma cell lines varied greatly in the ATase level from 0 to 212 fmol $mg^{-1}$ protein. Figure 1 shows the relationship between ATase activity and (A) $IC_{50}$ of BCNU and (B) cross-link index. The correlation coefficient between ATase and BCNU cytotoxicity was significant ($P$<0.001), higher levels of ATase (over 90 fmol $mg^{-1}$ protein) being associated with $IC_{50}$ values over five times as high as those in cell lines with undetectable ATase levels. There was also a strong correlation ($P$<0.001) between ATase and BCNU-induced cross-linking. The correlation coefficients for all the parameters are shown in Figure 2. There was no significant correlation ($P$=0.093) between cisplatin cytotoxicity and ATase activity or between cross-linking by cisplatin and ATase ($P$=0.9). Both drugs, however, showed a relationship between $IC_{50}$ and cross-linking ($P$<0.05). Cell lines having high levels of ATase were thus resistant to BCNU and had few DNA interstrand cross-links. One cell line (478) did not conform to this distribution with an ATase of 21 fmol $mg^{-1}$ protein and a high level of DNA-ISC but was relatively resistant to the cytotoxic effect of BCNU. Finally, a weaker association of cellular resistance ($IC_{50}$ value) was observed between the two drugs ($P$<0.05) by comparing $IC_{50}$ values. Figure 2 relates to tests for the pairwise association of variables. In addition to these, multiple linear regression analysis was used in an attempt to find dependencies of each of the variables on

© Cancer Research Campaign 1997

**Figure 3** Relationship in sublines between ATase level (a-0, b-53, c-146, d-155 fmol mg$^{-1}$ protein) and (**A**) $IC_{50}$ of BCNU, (**B**) $IC_{50}$ of cisplatin, (**C**) cross-link index at 50 μM BCNU and (**D**) cross-link index at 20 μM cisplatin combinations of the other variables, however no consistent pattern could be discerned.

Sublines were derived from line SB, which varied in ATase from 0 to 155 fmol mg$^{-1}$ protein. There was a clear relationship between increased ATase level and increased resistance (Figure 3A) and decreasing DNA-ISC (Figure 3C), following treatment with BCNU. In contrast, the sublines all remained relatively sensitive to treatment with cisplatin (Figure 3B), and all had a high level of DNA-ISC (Figure 3D).

### DISCUSSION

These results confirm that ATase levels vary in human glioma cell lines and that a high level of ATase correlates with resistance and decreased DNA-ISC formation in cell lines after treatment with BCNU. They also show a weak correlation between cellular resistance to BCNU and cisplatin.

Cell lines with ATase level greater than 25 fmol mg$^{-1}$ protein were more resistant to BCNU and had fewer DNA interstrand crosslinks induced by this agent. Once the level of ATase was greater than 90 fmol mg$^{-1}$ protein, the cell lines were generally very resistant and any increase in ATase above 90 fmol mg$^{-1}$ protein did not significantly increase resistance or decrease the number of DNA cross-links to BCNU. This suggests that there is a plateau level of ATase protection against DNA damage by CNUs, and above that level other mechanisms may be important. These results confirm previous studies (Aida et al, 1987; Sariban et al, 1987) that concluded that ATase is an important mechanism of resistance to CNUs in glioma lines and that high levels of ATase are associated with fewer DNA interstrand cross-links. Similar results have been reported from cell lines from a variety of malignant tumours (Robins et al, 1983; Brent, 1984; Bodell et al, 1986; Ludlum et al, 1986).

One cell line (478) did not conform to this distribution, and there is no explanation for this observation. This cell line also had a level of DNA-ISC induced by cisplatin out of proportion to its sensitivity and thus may lack a different DNA repair mechanism, thereby possessing an alternative mechanism of drug resistance.

This study has more precisely established the relationship of ATase level to CNU and another DNA-binding drug, cisplatin. There was no significant correlation observed for ATase in relation to cytotoxicity or DNA-interstrand cross-links induced by cisplatin; this was not expected as cisplatin forms DNA interstrand cross-links through the $N^7$ position of guanine (Eastman, 1982). There was, however, a correlation between sensitivities to BCNU and cisplatin, which implies that there may be an additional and associated mechanism of resistance. The results in the sublines of the cell line SB would suggest that this was not mediated through ATase as they showed excellent correlation between increasing ATase and increasing resistance and DNA-ISC after treatment with BCNU but showed little variation in resistance or DNA-ISC induced after treatment with cisplatin. Interestingly, however, the SB line, in contrast to the trend with other cell lines with high ATase, was very sensitive to cisplatin and had a corresponding high level of DNA-ISC induced by this agent. If this cell line is omitted from the statistical analysis the $IC_{50}$ for cisplatin becomes strongly correlated with ATase ($P < 0.01$) in the other 14 lines.

Other investigators have not simultaneously correlated DNA-ISC, cytotoxicity and ATase in cisplatin- and CNU-treated cells. One report examined 13 human glioma cell strains and found that there was a weak but significant correlation between DNA-ISC induced by cisplatin and BCNU. Two of the cell lines were assessed for sensitivity to BCNU, which showed that the MER +ve cell line was more resistant to CNU than the MER –ve cell line. No cell lines were examined for sensitivity to any other alkylating agent (Sariban et al, 1987). In contrast, another study examined five well-established human glioma cell lines and also found high ATase levels correlated with resistance and decreased DNA-ISC to CNU, but, in cell lines with intermediate resistance or sensitivity, the level of DNA-ISC was the same. They concluded that there was no correlation between CNU and cisplatin in cell kill, number of sister chromatid exchanges induced and degree of DNA-ISC. Closer examination of these results shows that all the cell lines had a similar number of cross-links formed after treatment with BCNU, except for the one line that was resistant, which had fewer crosslinks. All the cell lines had a similar cytotoxicity, DNA-ISC and sister chromatid exchanges after treatment with cisplatin (Aida et al, 1987). A further series examined ten different human cell lines and found no statistically significant correlation between cytotoxicity to cisplatin or DNA-ISC induced by cisplatin and Mer phenotype. There appeared to be a trend towards Mer +ve cell lines being more resistant to cisplatin. These results are similar to our findings, but cytotoxicity to cisplatin was not correlated with cytotoxicity to a nitrosourea (Laurent et al, 1981). Another study investigated four rat brain tumour cell lines and found that resistance to BCNU was associated with decreased cross-link formation. In these cell lines, cytotoxicity and the number of DNA cross-links formed after treatment with cisplatin were similar. The cell lines used in this experiment were cultured after exposure to CNUs and thus may have selected out cells with resistant mechanisms specific for CNUs, similar to the sublines in our study (Bodell et al, 1985). Indirect evidence that showed that *N*-methyl-*N'*-nitro-*N*-nitrosoguanidine pretreatment before cisplatin did not increase cytotoxicity or DNA-ISC led the authors to conclude that there was no cross-resistance between CNUs and cisplatin; however, they did not compare the relative sensitivities and ability to induce DNA-ISC of the two agents (Gibson et al, 1985). In support of our study, it has been shown that novobicin pretreatment to inhibit topoisomerase II activity has decreased the rate of repair of both cisplatin- and BCNU-induced DNA interstrand cross-links, with a corresponding increase in cytotoxicity. This implies that a DNA repair mechanism is involved in resistance to both these agents (Ali-Osman et al, 1993). More recently, a report of preliminary data showing response to cisplatin and cyclophosphamide in patients with ovarian carcinoma showed correlation with a low ATase level (Chen et al, 1994). This association, together with our results, suggests that there may be an additional resistance mechanism associated with ATase expression which confers resistance to cisplatin.

Although the relationship between resistance to BCNU and cellular activity of ATase has been shown previously, there was still uncertainty as to how important ATase is as a mechanism of resistance. This study indicates that ATase is the major mechanism of resistance to BCNU in glioma cells. It has also demonstrated cross-resistance between BCNU and cisplatin that is not related to ATase, implying that there may be an additional and associated mechanism of resistance.

### ACKNOWLEDGEMENTS

This project was supported by the Cancer Research Campaign (Grant SP1552/0701). JB acknowledges the Cancer Research

© Cancer Research Campaign 1997

Campaign for a Clinical Research Training Fellowship in Medical Oncology. Paul Nicholson is thanked for the statistical analysis.

### REFERENCES

Aida T and Bodell WJ (1987) Cellular resistance to chloroethylnitrosoureas, nitrogen mustard, and cis-diamminedichloroplatinum (II) in human glial-derived cell lines. *Cancer Res* **47**:1361–1366

Ali-Osman F, Berger MS, Rajagopal S, Spence A and Livingston RB (1993) Topoismerase II inhibition and altered kinetics of formation and repair of nitrosourea and cisplatin-induced DNA interstrand cross-links and cytotoxicity in human glioblastoma cells. *Cancer Res* **53**: 5663–5668

Bodell WJ, Gerosa M, Aida T, Berger MS and Rosenblum ML (1985) Investigation of resistance to DNA crosslinking agents in 9L cell lines with different sensitivities to chloroethylnitrosoureas. *Cancer Res* **45**:3460–3464

Bodell WJ, Aida T, Berger MS and Rosenblum ML (1986) Increased repair of $O^6$-alkylguanine DNA adducts in glioma-derived human cells resistant to the cytotoxic and cytogenetic affects of 1,3-bis(2-chloroethyl)-1-nitrosourea. *Carcinogenesis* **7**:879–883

Brent TP (1984) Suppression of cross-link formation in chloroethylnitrosourea-treated DNA by an activity in extracts of human leukemic lymphoblasts. *Cancer Res* **44**:1887–1892

Chen SS, Citron MC, Spiegel G and Yarosh D (1994) $O^6$-methylguanine-DNA methyltransferase in ovarian malignancy and its correlation with postoperative response to chemotherapy. *Gynecol Oncol* **52**: 175–179

Eastman A (1982) Separation and characterization of products resulting from the reaction of cis-diamminedichloroplatinum (II) with deoxyribonucleosides. *Biochemistry* **21**: 6732–6736

Erickson LC, Laurent G, Sharkey NA, and Kohn KW (1980) DNA crosslinking and monoadduct repair in nitrosourea-treated human tumour cells. *Nature* **228**: 727–729

Ewig RAG and Kohn KW (1978) DNA protein crosslinking and DNA interstrand crosslinking by haloethylnitrosoureas in L1210 cells. *Cancer Res* **38**: 3197–3203

Fine HA, Dear KBG, Loeffler JS, Black PM and Canellos GP (1993) Meta-analysis of radiation therapy with and without adjuvant chemotherapy for malignant gliomas in adults. *Cancer* **71**: 2585–2597

Gibson NW, Zlotogorski C and Erickson LC (1985) Specific DNA repair mechanisms may protect some human tumor cells from DNA interstrand crosslinking by chloroethylnitrosoureas but not from crosslinking by other anti-tumor alkylating agents. *Carcinogenesis* **6**: 445–450

Gonzaga PE and Brent TP (1989) Affinity purification and characterization of human $O^6$-alkylguanine-DNA alkyltransferase complexed with BCNU-treated, synthetic oligonucleotide. *Nucleic Acids Res* **17**: 6581–6590

Kohn KW (1977) Interstrand crosslinking of DNA by 1,3-bis(2-chloroethyl)-1-nitrosourea and other 1-(2-haloethyl)-1-nitrosoureas *Cancer Res* **37**: 1450–1454

Kohn KW (1981) Measurements of strand breaks and cross-links by alkaline elution. In *DNA Repair. A Laboratory Manual of Research Procedures*, Vol. 1, Part B, Friedberg EC and Hanawalt PC (eds), pp. 379–401. Marcel Dekker: New York

Laurent G, Erickson LC, Sharkey NA and Kohn KW (1981) DNA cross-linking and cytotoxicity induced by cis-diamminadichloroplatinum(II) in human normal and tumour cell lines. *Cancer Res* **41**: 3347–3351

Lippert B (1981) Effects on $N^7$ platinum binding on the hydrogen- bonding behavior of 9-ethylguanine. *J Amer Chem Soc* **103**: 5691

Ludlum DB, Mehta JR and Tong WP (1986) Prevention of 1-(3- deoxycytidyl), 2-(1-deoxyguanosinyl)ethane cross-link formation in DNA by rat liver $O^6$-alkylguanine-DNA alkyltransferase. *Cancer Res* **46**: 3353–3357

Robins P, Harris A, Goldsmith I and Lindahl T (1983) Cross-linking of DNA induced by chloroethyl-nitrosourea is prevented by $O^6$-methylguanine-DNA methyltransferase. *Nucleic Acids Res* **11**: 7743–7758

Rubinstein LV, Shoemaker RH, Paull KD, Simon RM, Tosini S, Skelan P, Scudiero DA, Monks A and Boyd MR (1990) Comparison of *in vitro* anticancer-drug-screening data generated with a tetrazolium assay versus a protein assay against a diverse panel of human tumour cell lines. *J Natl Cancer Inst* **82**: 1113–1118

Sariban E, Kohn KW, Zlotogorski C, Laurent G, D'Incalci M, Day R, Smith BH, Kornblith PL and Erikson LC (1987) DNA cross-linking responses of human malignant glioma cell strains to chloroethylnitrosoureas, cisplatin, and diaziquone. *Cancer Res* **47**: 3988–3994

Tong WP, Kirk MC and Ludlum DB (1982) Formation of the cross-link 1[N(3)-deoxycytidyl], 2-[N(1)-deoxyguanosinyl]ethane in DNA treated with N,N-bis(chloroethyl)-N-nitrosourea. *Cancer Res* **42**: 3102–3105

© Cancer Research Campaign 1997